# Insight, Neurocognition, and Schizophrenia: Predictive Value of the Wisconsin Card Sorting Test

**DOI:** 10.1155/2013/696125

**Published:** 2013-11-04

**Authors:** John Stratton, Philip T. Yanos, Paul Lysaker

**Affiliations:** ^1^Northwestern University, Feinberg School of Medicine, Chicago, IL 60611, USA; ^2^John Jay College of Criminal Justice, City University of New York, New York, NY 10019, USA; ^3^Roudebush VA Medical Center, Indianapolis, IN 46202, USA; ^4^Indiana University School of Medicine, Indianapolis, IN 46202, USA

## Abstract

Lack of insight in schizophrenia is a key feature of the illness and is associated with both positive and negative clinical outcomes. Previous research supports that neurocognitive dysfunction is related to lack of insight, but studies have not examined how neurocognition relates to change in insight over time. Therefore, the current study sought to understand how performance on the Wisconsin Card Sorting Test (WCST) differed between participants with varying degrees of change in insight over a 6-month period. Fifty-two patients with schizophrenia or schizoaffective disorder were administered the WCST and Positive and Negative Syndrome Scale (PANSS) at baseline, and the PANSS was again administered at a 6-month follow-up assessment. Results indicated that while neurocognition was related to insight at baseline, it was not related to subsequent change in insight. The implications of findings for conceptualization and assessment of insight are discussed.

## 1. Introduction

Lack of insight into one's illness has been cited as common among people with psychotic disorders and in particular schizophrenia. Lack of insight can be manifested in the denial or lack of awareness of the presence or impact of symptoms or the need for treatment [1]. Those with schizophrenia have been found to have higher levels of impaired insight in comparison to other psychotic disorders [2–6], with some studies citing this as the most common clinical phenomenon linked with schizophrenia [7, 8]. From a broader perspective, lack of insight reflects not so much the absence of a single piece of knowledge but a failure to construct an adaptive narrative account of the challenges posed by having a psychiatric illness [9, 10]. 

Although not a diagnostic criterion, lack of insight has become an important topic in the schizophrenia literature due to its utility as a prognosis indicator [11, 12] and potential marker of a subtype of the illness [13]. A better understanding of the etiological correlates of insight could enhance treatment modalities [11], improve prevention, and elucidate the relationship between insight and other clinical symptoms [14]. A number of such associations have already been found, including correlations between poor insight and severity of psychopathology [4, 15, 16], increased number of hospitalizations [4, 15], violent behavior [17, 18], lower treatment adherence [4, 19, 20], and poor premorbid or present adjustment [21]. Despite recognition as an important concept within behavioral science, reaching consensus as to what constitutes insight has proved challenging. Initially, insight was considered a dichotomous construct that was either present or absent and was measured by a patient's verbal recognition of existing psychological difficulties [22]. This conceptualization, however, has evolved into one of multidimensionality, where insight incorporates a multitude of phenomena [23], and is measured on a continuum. With this multidimensional approach have come a variety of new definitions. Such definitions include a combination of any number of the following dimensions: acceptance of the illness label, awareness of having a mental disorder, perceived need for treatment, awareness of treatment benefit, attribution of benefits to treatment, awareness of signs and symptoms, awareness of social consequences of illness, attribution of signs and symptoms to having a mental disorder, and lack of judgment [24]. Additionally, it has been argued that, beyond these dimensions, ethnicity, culture, interpersonal factors, education, social class, or intellectual functioning plays important roles in insight [24]. Despite inconsistent definitions across studies, there are a number of common components to the majority of definitions, with most including awareness of having a mental disorder and awareness and attribution of symptoms [22, 24–27]. 

As a result of the inconsistency in defining insight, there is a lack of continuity in how the construct is measured an inconsistency used by some to explain contradictory findings within the insight and the psychosis literature [28]. In part, the difficulty of defining and measuring insight is explained by one's theoretical orientation regarding the origins of impaired insight [24]. Existing theories attribute insight to positive, negative, and disorganized symptoms [1, 29], to defense mechanisms [30–33], and to culturally dependent and socially constructed views of insight [24].

Those utilizing a neurological framework for understanding impaired insight hypothesize that, like those suffering from anosognosia (a clinical syndrome in which neurological disturbances are denied), lack of insight in psychosis results from abnormalities in neuroanatomical structures [34]. Similarly, those conceptualizing insight as a result of neurocognitive dysfunction suggest that neurocognitive impairment decreases one's capacity to construct a personal narrative for understanding one's experience with illness, a concept that is thought to be involved in insight [9, 10].

Within the neurocognitive dysfunction literature, discrepant results have been found regarding the association between such dysfunction and diminished insight [15, 35–37]. Like the broader area of neurocognition and insight, studies investigating executive functioning and diminished insight have produced contradictory findings. Despite lacking strong consensus, studies have, with some consistency, found cross-sectional associations between impaired insight and the WCST. Specifically, previous research indicates that categories completed and perseverative errors from the WCST are significantly, albeit modestly, associated with insight [38–44]. Some, however, have failed to replicate such findings [26, 45–48]. 

In 2006, a meta-analysis, conducted by Aleman et al. [35], included 35 studies investigating the relationship between various insight measures and five cognitive domains (total cognition, IQ only, memory, frontal executive function, and performance on the WCST). Results from this study indicated a significant cross-sectional relationship between insight and the WCST, with an effect size of *r* = 0.23. Aleman et al. [35] noted the unique role perseveration errors played in impaired insight. This conclusion proffered by Aleman et al. [35] is found elsewhere within the literature [23, 43, 49].

While there appears a modest cross-sectional relationship between the WCST and insight, contradictory results have made the nature of this relationship unclear. A major limitation of prior studies is that they have not examined whether the WCST predicts change in insight over time. A better understanding of the prospective relationship between WCST (specifically perseveration errors) and insight could provide useful information about the trajectory of patients with impaired insight. Therefore, the current study aimed to assess how performance on neurocognitive measures differed between participants with varying degrees of insight over a 6-month period. It was hypothesized that those with worsening insight would show more neurocognitive dysfunction than those who remained stable or improved in insight.

## 2. Materials and Methods

Participants were 52 patients (51 male, 1 female) receiving outpatient services from either a midwestern psychiatric outpatient VA clinic or a local CMHC. Participants received a DSM-IV diagnosis of schizophrenia (*n* = 38) or schizoaffective disorder (*n* = 14). The mean number of lifetime hospitalizations was 7.8 (*sd*⁡ = 7.90) with an average first occurrence at age 27 (*sd*⁡ = 6.34). The mean age was 47.2 (*sd*⁡ = 9.01). Mean education was 13.8 (*sd*⁡ = 4.29). Participants were primarily White (*n* = 34) and African American (*n* = 17), with one Latino. No participants had been hospitalized, changed medication, or changed housing in the month prior to enrolling in the study. Participants with active substance dependence or intellectual disability were excluded from participation.

Clinical symptoms of schizophrenia were assessed using the PANSS [50, 51]. The scale is composed of 30 items and is used widely in clinical and research settings. It is regarded as a reliable tool for symptom assessment [52]. In accordance with previous research on the factor structure of the PANSS [53, 54], the current study utilized the five-factor model (hostility, emotional discomfort, positive and negative symptoms, and cognition) in assessing clinical symptoms of schizophrenia [55].

 Insight was assessed using the PANSS item G12. G12 is a global clinical assessment of lack of judgment and insight. It measures one's level of insight by assessing one's ability to recognize psychiatric illness, need for treatment, decision-making, and planning.

 Executive functioning was assessed using the WCST. The WCST was developed by Grant and Berg in 1948 [56] and is thought to measure preservation, set shifting, and abstract thinking [56, 57]. The WCST is a common measure of executive functioning within the neuropsychological literature [58–60].

 Participants were recruited from VA Medical Center's comprehensive day hospital where they were receiving outpatient treatment. Participants were in a postacute or stable phase of their disorder (no hospitalizations or alterations in their medication or housing within the last month). Through chart reviews, individuals identified as having a history of mental retardation were excluded from the study. Once informed consent was obtained, a clinical psychologist confirmed diagnoses using the Structured Clinical Interview from DSM-IV (SCID) [61]. Participants were then given the WCST and PANSS at baseline and the PANSS again at a 6-month follow-up assessment.

Initially, a correlation matrix was created using the variables of insight (change scores), insight at baseline, WCST (perseveration errors, categories completed) demographics, and total scores of the PANSS five factors (positive symptoms, negative symptoms, cognition, hostility, and emotional discomfort; the cognition total score did not include the G12 item as this was used to assess insight). Insight change scores were computed prior to analysis by subtracting baseline from endpoint insight scores. Any variable found to correlate significantly with insight was then entered into a multiple regression analysis. To account for baseline insight scores, insight change scores were entered as the dependent variable.

 To examine if different patterns would appear if insight change was assessed categorically, participants were grouped into one of 3 groups based on their insight change scores: those that improved in insight, those that declined in insight, and those that did not change. A MANOVA was then conducted to assess whether those with worsening insight over 6-months demonstrate more neurocognitive dysfunction when compared to those with improved or static insight. WCST variables (categories completed, perseverative responses) and change scores from the PANSS five factors were entered as outcome variables. PANSS change scores were created by subtracting endpoint from baseline factor scores for the following factors: positive, negative, cognition (excluding G12), hostility, and emotional discomfort.

## 3. Results

Table 1 shows correlations between variables of interest (change in insight, baseline insight, PANSS factors, and WCST). As this table indicates, no variables were significantly correlated with change in insight. The PANSS cognitive factor was most strongly correlated with baseline insight. WCST categories completed had a more modest, yet significant, negative correlation with baseline insight. 

 Since no significant relationships were found between change in insight and other variables, a regression analysis predicting change in insight was not preformed. However, based on the relationships found in the correlation matrix, a multiple regression predicting baseline insight was conducted, with WCST categories completed, the PANSS cognitive factor, perseveration errors, and the PANSS negative factor entered as predictors. Perseveration errors and the PANSS negative factor were included in this regression analysis since they approached significant correlations with baseline insight. Results indicated that the model significantly predicted baseline insight (*r* ^2^ = .378, *F*(4, 46) = 6.998, *P* < .001). Of the predictors, only the PANSS cognitive factor significantly contributed to the model (beta = .575, *P* < .001).

 Participants were then grouped into categories based on whether their insight score increased, decreased, or did not change between baseline and 6 months. 10 participants (20%) were categorized as improved, 8 as decreased (15%), and 33 (65%) were unchanged. Mean WCST perseverative responses was 41.5 (*sd*⁡ = 33.3) for the improved insight group, 42.75 (*sd*⁡ = 36.75) for the declined insight group, and 38.94 (*sd*⁡ = 28.67) for the unchanged insight group. Mean WCST categories completed for the improved, declined, and unchanged insight groups were 3.1 (*sd*⁡ = 1.97), 3.5 (*sd*⁡ = 2.27), and 3.3 (*sd*⁡ = 2.2), respectively. To assess if the WCST or PANSS scores differed between these 3 groups, a MANOVA was conducted, which failed to demonstrate a statistically significant difference between groups on any of the outcome variables (*F*(14, 86) = 1.12, *P* > .05). 

## 4. Discussion

 Neither the PANSS cognitive factor nor WCST variables were found to statistically significantly predict change in insight, thus failing to support the current study's hypothesis. Similarly, a MANOVA failed to find group differences on WCST variables between insight trajectories. These findings indicate that neurocognition remains similar across insight trajectories. One possible interpretation of these findings is that neurocognitive functioning, and specifically the capacity for cognitive flexibility, is unassociated with insight trajectory and that other factors would account for insight change. In addition, the regression analysis predicting baseline insight indicated that, despite a significant correlation between WCST perseveration errors and baseline insight, WCST perseveration errors did not significantly contribute to the prediction of insight when other factors were included in the equation. While perseveration errors have been found to be predictive of insight by others [43, 49], the predictive value of perseveration went unsupported in this study, a conclusion also drawn by Collins et al. [46] and Rossell et al. [62]. Like perseveration errors, categories completed also failed to predict baseline insight. Of those studies assessing whether categories completed predict insight, most have found this variable to have little to no predictive value [39, 41, 62]. 

 An alternative explanation for finding a lack of relationship between the WCST and insight change is the lack of sensitivity of the current study's insight measure. While the PANSS item G12 measures the concepts of awareness of illness, illness severity, and need for treatment, this item could simply be measuring fluctuations in agreement or rejection of symptoms/labels and failing to capture aspects of insight that some argue to be central to the construct (i.e., capacity to construct a personal narrative of illness experience that can be understood by others). Therefore, it is possible that insight, as measured by the PANSS, functions independently of neurocognition. Perhaps a more sensitive measure of insight, like that proposed by Lysaker et al. [9, 10], would better capture the relationship between neurocognitive dysfunction and changes in insight over time. Indeed, the capacity to construct a personal narrative would be diminished by problems in forming concepts, profiting from correction, and conceptual flexibility (perseveration errors). It is presumable that the capacity for abstract thinking, change and refinement of a narrative, and integration of feedback from others would aid in the construction of a coherent and understandable narrative.

 Despite adding to the literature by assessing the prospective relationship between neurocognition and insight, the current study had a number of limitations. First, the sample size was small, restricting statistical power. Although a smaller sample size is not uncommon within this vein of research [41, 62], a larger sample would provide a better understanding of the relationships between the current study's variables and allow more confident interpretations of these relationships. Second, change in insight groups was small; therefore, the lack of findings could be the result of a restriction in range for the insight variable. Third, insight was measured using the G12 item of the PANSS. Although this measure of insight is common within the literature, an independent insight scale would have provided greater confidence in interpreting the relationship between insight and the PANSS cognitive factor. Using a measure of insight that facilitated analyzing various components of insight (i.e., current awareness of symptoms, past awareness of symptoms) could have provided a better understanding of how different aspects of insight relate to neurocognitive and clinical variables. Fourth, assessing WCST at multiple points in time would have provided further opportunity to understand the relationship between WCST and insight. Finally, participants in this study were relatively stable outpatients. Investigating insight in a first episode or inpatient sample may have provided a greater degree of variance in insight and symptom severity.

 Future research should investigate neurocognitive difference between insight trajectories using a multidimensional measure of insight, as opposed to a unidimensional measure. Specifically, assessing whether WCST (perseveration errors or categories completed) relates to various aspects of insight (rather than insight as a whole) could shed light on the nature of the insight-neurocognition relationship. Utilizing a measure of insight that incorporates a narrative approach could prove useful in understanding how neurocognitive dysfunction, and perseveration errors in particular, affects one's ability for narrative construction. One possibility that remains unexplored is that poorer neurocognition could lead to an inability to construct a personal narrative that provides an alternate understanding of illness experiences (e.g., one that does not involve mental illness). This inability to construct a personal narrative could actually result in higher superficial insight (e.g., shallow agreement with the label but no in-depth understanding) for individuals with diminished neurocognition as they would be unable to provide alternative accounts of their symptoms (see Roe et al. [63], for a discussion of how narrative insight can be independent from more superficial insight). This could ultimately lead to greater denial so as to avoid the negative stigma attached to identification with mental illness.

## 5. Conclusions

 Findings from the current study lend mixed support for the relationship between neurocognition and insight. While WCST was not found to be predictive of insight, the PANSS cognitive factor significantly contributed to the regression model, suggesting a relationship between insight and a more general measure of cognition. These findings suggest that performance on the WCST fails to delineate between insight trajectories, when insight is conceptualized as need for treatment, illness awareness, and illness severity. A narrative approach to understanding insight may help in elucidating how neurocognition relates to insight trajectory, as conceptual flexibility, concept formation, and benefitting from feedback likely contribute to the capacity to construct a personal narrative.

## Figures and Tables

**Table 1:** Correlation matrix of neurocognition, insight, and clinical variables.

	1	2	3	4	5	6	7
(1) WCST persev							
(2) WCST categories	−0.78**						
(3) PANSS positive	0.16	−0.33*					
(4) PANSS negative	0.23	−0.16	0.31*				
(5) PANSS hostility	0.08	−0.04	0.38**	−0.01			
(6) PANSS emotion	0.11	−0.1	0.5**	0.27	0.28*		
(7) PANSS cognition	0.33*	−0.33*	0.45**	0.46**	0.28*	0.1	
(8) Insight baseline	0.25	−0.31*	0.09	0.26	0.23	−0.18	0.6**
(9) Insight change	0.01	0.06	0.01	−0.14	−0.13	−0.02	0.05

*Correlation is significant at the 0.05 level (2-tailed).

**Correlation is significant at the 0.01 level (2-tailed).
